# An Improved Strategy to Recover Large Fragments of Functional Human Neutrophil Extracellular Traps

**DOI:** 10.3389/fimmu.2013.00166

**Published:** 2013-06-24

**Authors:** Lorena Barrientos, Viviana Marin-Esteban, Luc de Chaisemartin, Vanessa Lievin Le-Moal, Catherine Sandré, Elsa Bianchini, Valerie Nicolas, Marc Pallardy, Sylvie Chollet-Martin

**Affiliations:** ^1^INSERM, UMR-S 996, “Cytokines, Chemokines and Immunopathology”, UniverSud, Paris, France; ^2^Faculté de Pharmacie, UniverSud, Paris, France; ^3^AP-HP, Groupe Hospitalier Paris Nord Val de Seine, Hôpital Bichat, Unité d’Immunologie «Auto-immunité et Hypersensibilités», Paris, France; ^4^CNRS, UMR 8076 (BioCIS), Equipe «chimiothérapie antiparasitaire», Faculté de Pharmacie, UniverSud, Paris, France; ^5^Laboratoire d’hématologie, EA 4531, Faculté de Pharmacie, UniverSud, Paris, France; ^6^IFR 141, IPSIT «Institut Paris-Sud d’Innovation Thérapeutique», Plateforme Imagerie Cellulaire, Faculté de Pharmacie, Paris, France

**Keywords:** neutrophil, netosis, neutrophil extracellular traps, isolation, quantification, characterization, microbicidal activity

## Abstract

Netosis is a recently described neutrophil function that leads to the release of neutrophil extracellular traps (NETs) in response to various stimuli. NETs are filaments of decondensed chromatin associated with granular proteins. In addition to their role against microorganisms, NETs have been implicated in autoimmunity, thrombosis, and tissue injury. Access to a standardized source of isolated NETs is needed to better analyze the roles of NETs. The aim of this study was to develop a procedure yielding soluble, well-characterized NET preparations from fresh human neutrophils. The calcium ionophore A23187 was chosen to induce netosis, and the restriction enzyme *Alu*I was used to prepare large NET fragments. DNA and proteins were detected by electrophoresis and specific labeling. Some NET proteins [histone 3, lactoferrin (LF)] were quantified by western blotting, and double-stranded DNA (dsDNA) was quantified by immunofluorescence. Co-existence of dsDNA and neutrophil proteins confirmed the quality of the NET preparations. Their biological activity was checked by measuring elastase (ELA) activity and bacterial killing against various strains. Interindividual differences in histone 3, LF, ELA, and dsDNA relative contents were observed in isolated NETs. However, the reproducibility of NET preparation and characterization was validated, suggesting that this interindividual variability was rather related to donor variation than to technical bias. This standardized protocol is suitable for producing, isolating, and quantifying functional NETs that could serve as a tool for studying NET effects on immune cells and tissues.

## Introduction

Polymorphonuclear neutrophils (PMN) are the most abundant white blood cells in humans. In addition to their crucial role in protecting against infections, recent evidence suggests that PMN are also key components of the effector and regulatory mechanisms of both innate and adaptive immune responses (Mantovani et al., 2011; Phillipson and Kubes, 2011). PMN are the first cells to migrate to sites of infection and sterile inflammation, and exhibit a wide range of sophisticated functions. A new function, netosis, was described in Brinkmann et al. (2004).

During netosis, PMN release neutrophil extracellular traps (NETs) that can entangle pathogens but also contribute to various inflammatory diseases. PMN undergo specific morphological changes during netosis, but the molecular mechanisms of DNA release are not yet fully understood. First, chromatin decondensation leads to the loss of the lobulated nucleus. Then, disintegration of intracellular membranes allows chromatin and extranuclear proteins to mix. The final step is the release into the extracellular medium of chromatin filaments decorated with PMN proteins derived from several cell compartments (Fuchs et al., 2007). Netosis is dependent on the oxidative burst, via NADPH-oxidase 2 (NOX-2) and rho small GTPase Rac2 activation (Bianchi et al., 2011; Lim et al., 2011), as well as on histone citrullination by peptidylarginine deiminase (PAD4) (Li et al., 2010; Neeli and Radic, 2012) and nuclear translocation of granular myeloperoxidase (MPO) and elastase (ELA) (Metzler et al., 2010; Papayannopoulos et al., 2010), Recently, dynamic observations *in vivo* showed that NET-bearing PMN are still able to crawl and to engage FcγRIIA to internalize immune complexes (Chen et al., 2012; Yipp et al., 2012). Eosinophils (Yousefi et al., 2012), basophils, mast cells (von Kockritz-Blickwede et al., 2008), and probably other cell types can also produce extracellular traps.

A proteomic study of NETs identified 24 different proteins. Histones accounted for more than 70% of total NET proteins, while ELA, calprotectin, and lactoferrin (LF) were the most abundant non-nuclear proteins, representing between 2 and 6% of total proteins (Urban et al., 2009). During the last 2 years, we and others have identified other proteins associated with NETs, such as pentraxin 3, β2 integrin, HMGB1, and LL37 (Jaillon et al., 2007; Garcia-Romo et al., 2011; Marin-Esteban et al., 2012). Some reports suggest that NET protein diversity depends on the priming agent and/or the stimulus that triggers netosis (Garcia-Romo et al., 2011).

The main known function of NETs is to trap and kill microbes. Two recent reviews (Brinkmann and Zychlinsky, 2012; Kaplan and Radic, 2012) concluded that a broad variety of pathogens can induce and/or be killed by NETs, including bacteria, fungi, protozoan parasites, and even viruses (including HIV1). However, proteins contained in NETs can also have detrimental effects on the host. Indeed, we have previously reported that NETs can damage intestinal epithelial cells (Marin-Esteban et al., 2012), while histones are known to damage endothelial tissue, particularly during systemic lupus erythematosus (SLE) and sepsis (Xu et al., 2009; Villanueva et al., 2012). One of the most exposed organs might be the lung, as NET-induced damage has been reported *in vivo* during asthma, adult respiratory distress syndrome, transfusion-related acute lung injury, and cystic fibrosis (Dubois et al., 2012; Cheng and Palaniyar, 2013). Another adverse effect of NETs is their ability to hyperactivate the coagulation system, leading to atherosclerosis and thrombosis (Doring et al., 2012; Fuchs et al., 2012), and also to sepsis via tissue factor exposure (Kambas et al., 2012).

A role of NETs in tolerance-breaking and induction of autoimmunity is very probable, particularly in SLE (Bouts et al., 2012) and small-vessel vasculitis (Garcia-Romo et al., 2011; Cui et al., 2012; Sangaletti et al., 2012; Villanueva et al., 2012). Indeed, NET release exposes cytoplasmic, granular, and nuclear self antigens to the immune system. The antigenicity of these components may be modified by post-translational modifications such as oxidation and citrullination, or by intermolecular associations (Metzler et al., 2010; Papayannopoulos et al., 2010; Kambas et al., 2012; Liu et al., 2012; Neeli and Radic, 2012). For instance, LL37 renders NET-associated DNA immunogenic, making it able to activate plasmacytoid dendritic cells and to release type 1 interferons (Garcia-Romo et al., 2011).

This short summary of the literature evidences that several questions are still open. In particular, the effects of NET components on host cells and tissues are complex and difficult to assess. Interestingly, there is growing evidence that NETs might allow sustained cross-talk between PMN components and other immune cells such as dendritic cells and lymphocytes (Sangaletti et al., 2012; Tillack et al., 2012). Access to a standardized source of isolated NETs is needed to further analyze their role in both normal and abnormal immune responses. A few approaches to NET preparation have already been described. One consists of NET digestion by DNase I, followed by acetone protein precipitation (Urban et al., 2009). Another consists of physical NET dissociation by vigorous agitation, followed by MNase or DNase I digestion, yielding small NETs containing both DNA and proteins (Liu et al., 2012; Saffarzadeh et al., 2012). However, some authors have reported that the microbicidal effect of NETs is lost after DNase treatment [due to double-stranded DNA (dsDNA) damage], and that small mono- or oligonucleotide structures are created by MNase treatment (Fuchs et al., 2007; Urban et al., 2009; Saffarzadeh et al., 2012).

Here we describe an alternative standardized procedure to isolate large soluble NETs from human PMN. Calcium ionophore A23187 was used to induce netosis, and the restriction enzyme *Alu*I was used to recover large heterogeneous NET fragments. Samples were standardized by quantifying four key NET components, namely dsDNA, histone H3 (H3), LF, and ELA. Isolated NETs exhibited biological activity, as shown in terms of ELA activity and bacterial killing.

## Materials and Methods

### Isolation of human blood polymorphonuclear neutrophils

Polymorphonuclear neutrophils were isolated from fresh buffy coats prepared from blood of healthy donors, provided by Etablissement Français du Sang (Rungis, France). First, leukocytes were separated from erythrocytes by sedimentation on a separating medium containing 5% Dextran T500^®^ (Pharmacia, Uppsala, Sweden) in 0.9% saline. PMN were separated from mononuclear cells by Ficoll centrifugation (*d* = 1.077 g/L, Eurobio, Les Ulis, France). Contaminating erythrocytes were removed by hypotonic lysis, and PMN were resuspended in Hank’s buffered salt solution (HBSS) (Gibco Life Technologies, Saint Aubin, France) supplemented with 0.05% fetal calf serum (FCS) (PAA, Les Mureaux, France) and 10 nM Hepes (Gibco).

### Time course of NET release after PMN stimulation with PMA or A23187

Freshly isolated PMN were immediately seeded in 96-well black plates (2 × 10^5^ cells/well) in the presence of 5 μM Sytox Green (Invitrogen, Saint Aubin, France), a non-cell-permeant DNA binding dye. Cells were then stimulated with increasing concentrations (2–250 nM) of phorbol 12-myristate 13-acetate (PMA) (Sigma Aldrich, Lyon, France) or the calcium ionophore A23187 (0.2–25 μM) (EMD chemicals, San Diego, CA, USA) at 37°C with 5% CO_2_ in the dark. DNA release was followed by measuring green fluorescence at 0, 60, 120, 180, and 240 min in a microplate fluorescence reader (TristarTM LB941 BERTHOLD, Bad Wildbad, Germany) at an excitation wavelength of 485 nm and an emission wavelength of 527 nm. All samples were tested in duplicate. These experiments allowed us to determine the optimal concentration of each stimulus, as well as the time of maximal NET release.

### Immunofluorescence labeling and observation of NETs

Freshly isolated PMN were seeded on polylysine-coated glass coverslips (2 × 10^5^ cells/well), allowed to settle, and then treated with 50 nM PMA or 5 μM A23187 at 37°C, as previously described (Marin-Esteban et al., 2012). After 3 h of activation, cells were fixed with 4% paraformaldehyde (PFA) (Electron Microscopy Sciences, Hartfield, PA, USA) at room temperature. Samples were then washed with phosphate buffered saline (PBS, Gibco Life Technologies), blocked with 2% bovine serum albumin (BSA) (PAA) and incubated with antibodies against ELA, LF (Sigma Aldrich), H3 (EMD chemicals), or citrullinated-histone H3 (cit-H3) (clone ab5103, Abcam, Cambridge, UK) for 30 min at room temperature, followed by Alexa Fluor 488 goat anti-rabbit IgG for 30 min (Molecular Probes, Karlsruhe, Germany). Isotype-matched antibodies were used as controls. The coverslips were mounted in medium containing 4′,6-diamidino-2-phenylindole (DAPI) (Southern Biotech, Birmingham, AL, USA) to label DNA. Cells were examined with an inverted epifluorescence microscope (AxioObserver Z1_Colibri, Zeiss, Germany) equipped with an MRm CCD camera. All images were acquired with a Plan-Apochromat 63X/1.4 NO oil immersion objective, using two light-emitting diodes (365 and 470 nm) for excitation and two bandpass filters (445/450 and 530/550 nm) to collect blue and green fluorescence, respectively.

### NET digestion by nucleases

In a first set of experiments, 4 μg of purified λDNA (Invitrogen) was treated with 4 U/mL DNase I (Sigma Aldrich), 4 U/mL MNase (New England BioLabs, France), or 4 U/mL *Alu*I (New England BioLabs) for 20 min at 37°C. The samples were then loaded on 0.8% agarose gels (w/v) prepared in Tris-acetate-EDTA buffer containing 0.5 μg/mL ethidium bromide (Invitrogen). Electrophoresis was run at 100 V for 30 min and DNA was visualized with an ultraviolet transilluminator (MiniBIS-Pro, DNR Bio-imaging Systems).

In a second set of experiments, A23187-stimulated PMN seeded in 12-well culture plates were treated with or without various concentrations of *Alu*I, DNaseI, or MNase (0.8, 4, and 20 U/mL) for 20 min at 37°C. The supernatant of each well was harvested and DNA was analyzed after electrophoresis, as described above.

### NET isolation

Freshly isolated PMN were seeded in 12-well culture plates (1.5 × 10^6^ cells/well) and stimulated with PMA (50 nM) or A23187 (5 μM). After 3 h at 37°C in the presence of 5% CO_2_, each well was carefully washed twice with 1 mL PBS and then treated with 400 μl of the restriction enzyme *Alu*I at 4 U/mL for 20 min at 37°C. The supernatant of each well was collected and centrifuged for 5 min at 300 × *g* at 4°C in order to remove whole cells and debris. The NET-rich supernatants were then characterized for their DNA and protein contents, using several approaches.

### DNA observation and quantification

The size of the recovered DNA fragments was estimated after electrophoresis as described above. DNA was quantified in NET samples by using Quant-iT™ PicoGreen^®^ dsDNA (Molecular Probes), an ultrasensitive fluorescent nucleic acid stain designed to quantify dsDNA in solution. The manufacturer’s instructions were followed, and calibration standards between 30 and 1000 ng/mL dsDNA were used. Fluorescence signals were measured in a microplate fluorescence reader (TristarTM LB941).

### Quantification of total protein, histone 3, and lactoferrin

Total NET protein was quantified with a bicinchoninic acid assay kit (Pierce, Rockford, IL, USA). Protein diversity was explored by SDS-PAGE. Proteins (30 μg of total protein) were separated in denaturing and reducing conditions by electrophoretic migration in 15% acrylamide gel, and then revealed by silver staining. To quantify specific NET proteins, 30 μg of total NET protein and various amounts of purified H3 (0.5–2 ng/μL) or LF (0.5–2 ng/μL) (Sigma Aldrich) were separated by electrophoresis in the same gel. Proteins were then transferred to Hybond membranes (Amersham Biosciences, Velizy-Villacoublay, France) overnight at 40 V and 4°C. After blocking with BSA, membranes were incubated with the antibodies against H3, cit-H3, and LF as described above. Primary antibody binding was detected with a goat anti-rabbit IgG antibody conjugated to horseradish peroxidase (Cell Signaling Technology, Danvers, MA, USA) and visualized by EZ-ECL (Biological Industries, Israel). Yields were calculated and H3 and LF levels were expressed as μg per 10^6^ PMN.

### Quantification of elastase activity

Elastase activity in NET samples was measured with an enzymatic kinetic method using the ELA substrate I (EMD chemicals) Boc-Ala-Ala-Pro-Ala-pNA (Boc = t-butoxycarbonyl; pNA = para-nitroanilide). NET solution and ELA substrate I at 222 μM in kinetic buffer (20 mM phosphate buffer, 0.15 M NaCl, 0.1 mM EDTA, and 0.1% PEG-8000, pH 7.4) were mixed at a volume ratio of 1:10 and the initial velocity of pNA release was recorded by measuring absorbance at 405 nm in a microplate reader. Various concentrations of purified ELA from human neutrophils (EMD chemicals) were used as standards. The reaction rate and corresponding ELA activity were determined using GraphPad^®^ software. Yields were calculated and H3 and LF levels were expressed as μg per 10^6^ PMN.

### Reproducibility testing

In order to validate our isolation technique, we assessed reproducibility testing. Fresh blood was obtained from four healthy donors. Each sample was immediately divided into three equal parts before PMN isolation. The NET-production protocol described above was then applied in order to get three NET samples for each donor. All the NET-rich supernatants were then characterized by assaying dsDNA, H3, LF, and ELA in triplicate as described above.

### Bacterial killing assay

Exponentially growing wild-type Afa/Dr DAEC strain C1845, wild-type *Salmonella enterica* serovar Typhimurium strain SL1344, wild-type *Shigella flexneri* strain M90T, and a clinical culture collection *Staphylococcus aureus* strains (Microbiology laboratory, Faculty of Pharmacy, Châtenay-Malabry, France) were subcultured in Luria–Bertani (LB) broth at 37°C for 18 h. Bacteria were washed, counted in a Salumbini chamber, and adjusted to 10^7^ colony forming units per mL (CFU/mL). Bacteria (100 μL) were incubated with isolated NETs at a final concentration of 50 ng/mL dsDNA for 45 min at 37°C. Samples were serially diluted, plated on LB agar, and incubated for 24 h at 37°C to determine the numbers of CFU/mL. Experiments were carried out in triplicate and repeated at least five times. Bacterial killing was determined as previously reported (Marin-Esteban et al., 2012).

### Statistical analysis

The data were analyzed with GraphPad Prism 5.01. The non-parametric Mann–Whitney test was used for comparisons. Differences with *p* values below 0.05 were considered significant. All data are expressed as mean ± SEM.

## Results

### Calcium ionophore A23187 induces netosis

**Figure 1:**
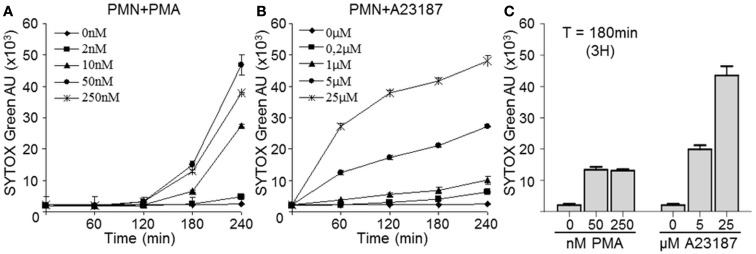
**Calcium ionophore A23187-activated PMN release dsDNA more rapidly than PMA-activated PMN**. PMN (2 × 10^5^) were incubated in the presence or absence of increasing concentrations of PMA **(A)** or A23197 **(B)** for 240 min. The presence of extracellular dsDNA was detected at the indicated times with Sytox Green. Both graphs are representative of three identical experiments and show means ± SEM of duplicate samples **(C)** Comparison of extracellular dsDNA levels 3 h after PMA or A23187 activation; data are means ± SEM (*n* = 3). AU, arbitrary units.

Netosis can be induced by various stimuli. PMA is generally used in the literature and was used here as a reference stimulus. In addition, we used calcium ionophore A23187, which is reported to be efficiently removed by washing steps (Betz and Henson, 1980). We compared the capacities of A23187 and PMA to induce human PMN netosis. NET release was monitored for 240 min by staining extracellular dsDNA with Sytox Green, a dye that is not incorporated by living cells. The green signal was weak and stable in resting PMN, while both PMA and A23187-induced dsDNA release in a time- and concentration-dependent manner, although with different kinetics (Figures 1A,B). With PMA concentrations of 10–250 nM, dsDNA release was detected after 120 min of incubation; at 250 nM PMA rather induced apoptosis as suggested by lower dsDNA levels and fragmented nuclei (not shown). With A23187 at 5 or 25 μM, extracellular dsDNA was detected after as little as 60 min. We therefore chose 3 h as the optimal time of stimulation. Moreover, after comparing the two stimuli at this time point (Figure 1C), we chose 5 μM A23187 and 50 nM PMA as the optimal concentrations for inducing comparable netosis. In these conditions we found a large amount of extracellular dsDNA and only a small number of apoptotic or necrotic nuclei (DAPI staining, data not shown).

**Figure 2:**
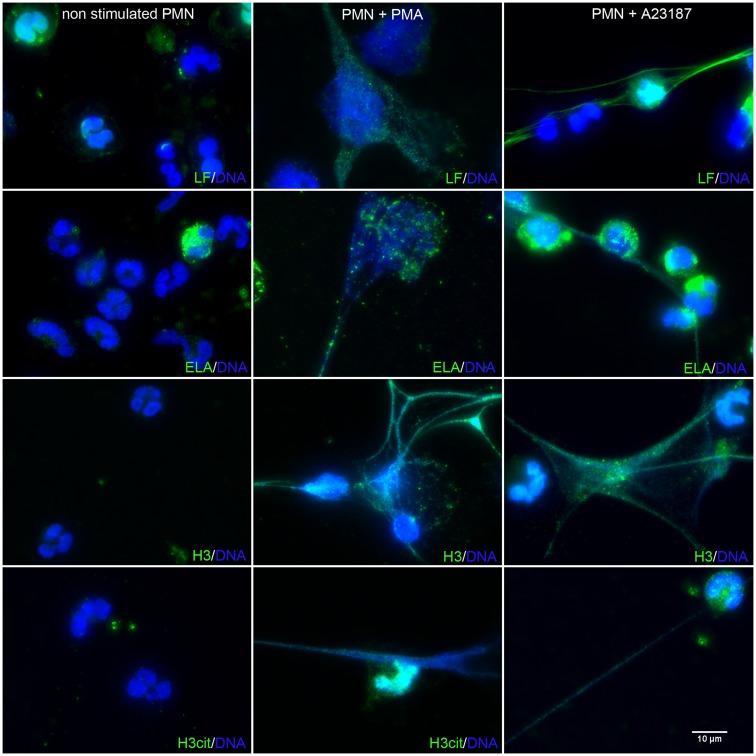
**A23187-stimulated PMN release NETs similar to those induced by PMA**. PMN were treated with PMA (50 nM) or A23187 (5 μM) for 3 h. NETs were observed by immunofluorescence microscopy after DNA staining with DAPI (blue), and after elastase, LF, H3, or cit-H3 staining with specific Abs followed by an Alexa Fluor 488-labeled secondary antibody (green). These experiments were repeated at least six times with PMN from different healthy controls.

To confirm that the isolated extracellular DNA corresponded to NET structures, we used immunolabeling of proteins known to be associated with NETs, as previously described (Marin-Esteban et al., 2012). Figure 2 shows resting PMN, A23187-activated PMN, and PMA-activated PMN. In the absence of stimuli, PMN did not expose any intracellular protein and exhibited an intact polylobulated nucleus, suggesting nuclear, and plasma membrane integrity. As expected, DAPI-stained extracellular DNA structures were observed in response to PMA or A23187. These cable- or web-like structures corresponded to cell chromatin as they carried histones, as shown by the presence of H3. Cit-H3 was also present, confirming its formation through netosis. Colocalization of extracellular DNA structures with LF and ELA, two granule-associated proteins, confirmed that these structures were NETs. Together, these results show for the first time that, in these conditions, A23187 activates netosis in human PMN as efficiently as PMA, leading to NET release.

### A23187-induced NETs can be digested by the restriction enzyme Alu-I

**Figure 3:**
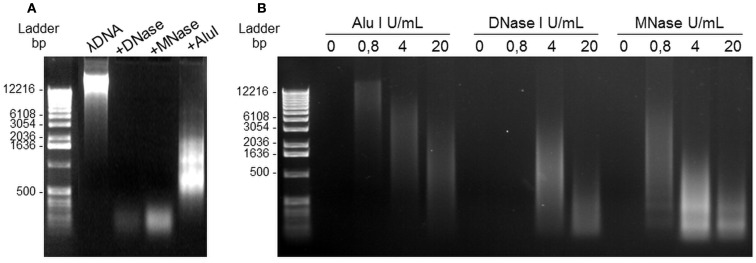
**DNA nucleases induce NET digestion**. **(A)** Migration profile of pure λDNA after digestion with 4 U/mL DNase, MNase, or Alu-I. **(B)** Alu-I, DNase, and MNase dose-effects on NET dsDNA obtained after A23187 stimulation of PMN. Incubation with the restriction enzymes lasted 20 min at 37°C. DNA migration took place in 0.8% agarose gel containing ethidium bromide.

The next step was to separate NETs from the cell body while maintaining their structure. In order to define the optimal conditions, the effects of several restriction enzymes on purified DNA (λDNA) were first compared. As shown in Figure 3A, *Alu*I yielded large heterogeneous DNA fragments, while DNaseI and MNase yielded smaller fragments. We then studied the concentration-response effect of these three enzymes on supernatants of A23187-stimulated PMN and confirmed that *Alu*I was optimal to get large heterogeneous fragments (>0.5 kbp), as compared with DNaseI and MNase (Figure 3B).

**Figure 4:**
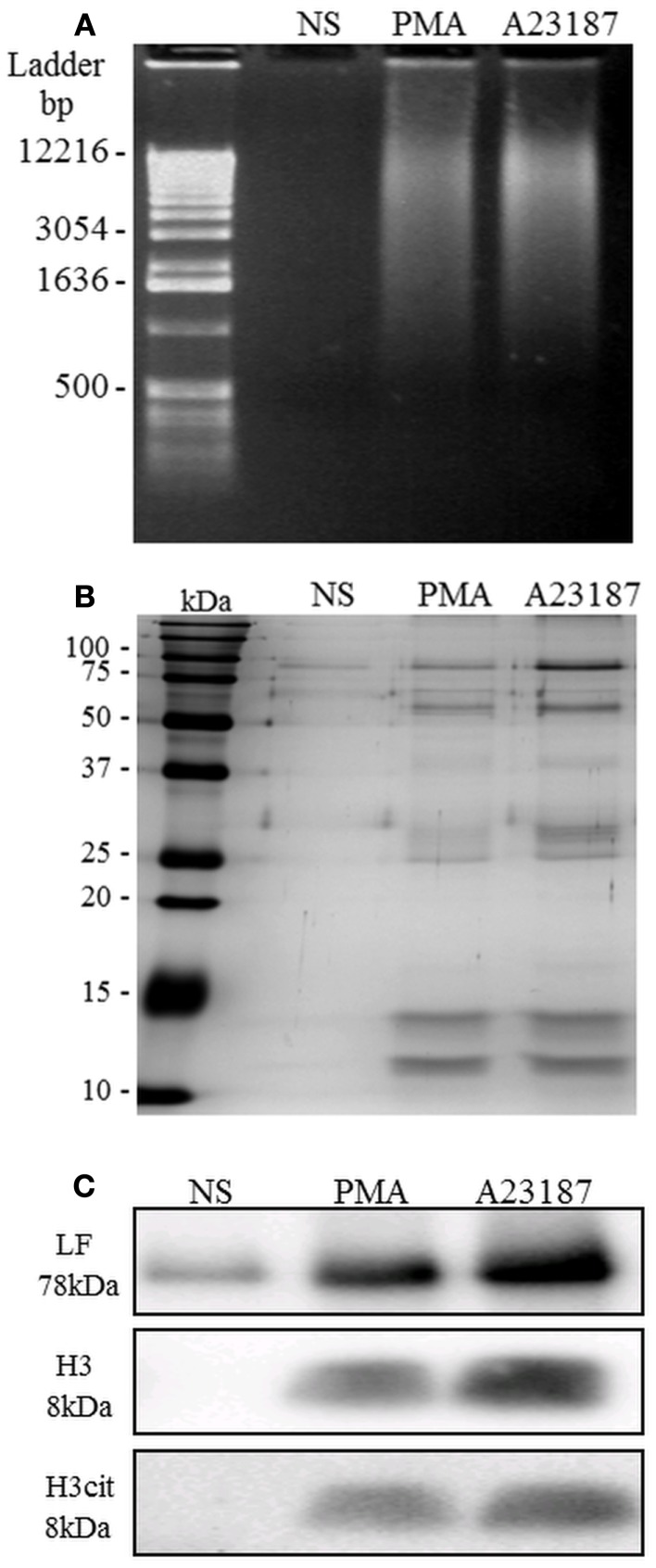
**NETs are recovered after AluI treatment of activated PMN**. Unstimulated PMN and PMA- or A23187-stimulated PMN were treated with the restriction enzyme Alu-I. **(A)** NET samples migrated on agarose gel were stained with ethidium bromide: dsDNA fragments were revealed as a smearing pattern along the gel. **(B)** Visualization of NET protein content by silver staining. Few proteins were observed in the untreated sample, whereas numerous proteins were observed in PMA- and A23187-NET samples, with similar profiles. **(C)** Identification of three specific NET proteins (LF, H3, and cit-H3) by immunoblotting. Cit-H3 is a signature of netosis. These experiments were repeated at least six times with PMN from different healthy controls.

NET-rich supernatants were then further analyzed. In supernatants from control resting PMN, *Alu*I treatment yielded no dsDNA, indicating that *Alu*I treatment itself induced no cell toxicity (Figure 4A). In contrast, in response to both stimuli, the samples contained a heterogeneous population of dsDNA fragments that migrated with a smearing pattern distributed from 0.5 kbp to more than 15 kbp. In these conditions the strongest signal corresponding to dsDNA fragments was comprised between 5 and 12 kbp (Figure 4A).

The protein composition of NETs was then evaluated by SDS-PAGE followed by silver staining (Figure 4B). Samples from resting PMN contained low concentrations of various proteins. In contrast, samples recovered after *Alu*I treatment of PMA- or A23187-stimulated PMN yielded stronger signals. The presence of LF, reported to be the second most abundant granule-derived protein on NETs (Urban et al., 2009), was confirmed by western blot (Figure 4C). The low level of LF in unstimulated PMN samples was probably due to basal cell degranulation. We also confirmed the presence of histone H3, a chromatin-associated protein, in A23187- or PMA-treated PMN but not in samples from resting PMN. Interestingly, citrullinated H3 was also present in NET samples, providing the signature of netosis.

Taken together, these results showed that this Alu-I-based approach yielded large soluble NET fragments composed of DNA and numerous neutrophil proteins.

### Interindividual differences in histone 3, LF, ELA, and dsDNA relative contents in isolated NETs

In order to better characterize NET composition and to standardize our protocol, we quantified four major constituents (LF, H3, dsDNA, and ELA) in NET samples obtained from 10 different healthy donors.

**Figure 5:**
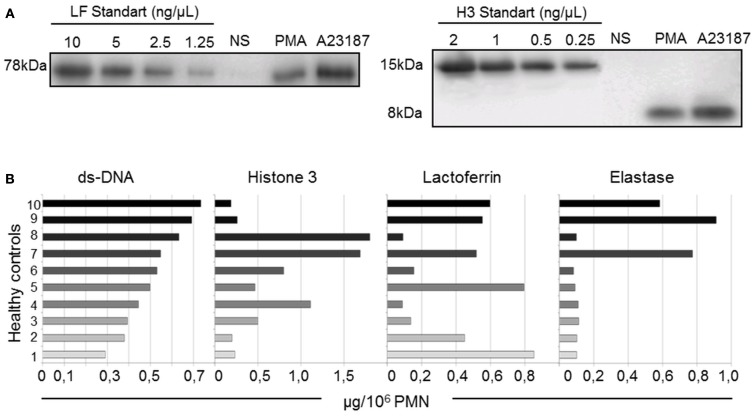
**Quantification of dsDNA and three NET-associated proteins shows interindividual heterogeneity (10 healthy controls)**. **(A)** Representative western blot of different concentrations of purified H3 and LF immunoblotted in parallel with isolated NETs and non-stimulated (NS) PMN supernatants from one healthy control. The bands were quantified by densitometry and plotted against the standard curve. **(B)** dsDNA, H3, LF, and ELA concentrations of A23187-NET samples from 10 healthy donors. Results are expressed in μg/10^6^ PMN.

We first assessed LF and H3 concentrations by quantitative immunoblot analysis. Figure 5A illustrates the results of a typical experiment with both standards and a NET sample from one donor. The apparent molecular weight of NET-associated LF and of purified LF were similar, whatever the netosis trigger. In contrast, NET-associated H3 displayed more rapid electrophoretic migration than purified H3, with an apparent loss of 7 kDa, suggesting partial H3 degradation. We also quantified dsDNA, widely used in the literature as a reliable marker of netosis (Urban et al., 2009). Finally, we showed the biological activity of these NETs by quantifying NET-associated ELA activity in an enzymatic test (Metzler et al., 2010; Papayannopoulos et al., 2010).

Despite the use of standardized protocols, the concentrations of these four components (expressed in μg per 10^6^ PMN, as described in Materials and Methods) differed across NETs from the 10 healthy controls (Figure 5B). The dsDNA content was the least variable parameter. Interestingly, the H3 content did not correlate with the dsDNA content, suggesting different degrees of chromatin decondensation or histone degradation in NETs from the different donors. LF and ELA levels did not correlate with dsDNA levels either. Importantly, 7 of the 10 NET preparations exhibited little ELA activity, whereas the 3 remaining samples showed high ELA activity. No statistically significant relationship was found between individual values of the four parameters in a given donor. This heterogeneity of NET composition might be related to interindividual differences in the netosis response to a given stimulus.

### The isolation NET technique is reproducible

**Figure 6:**
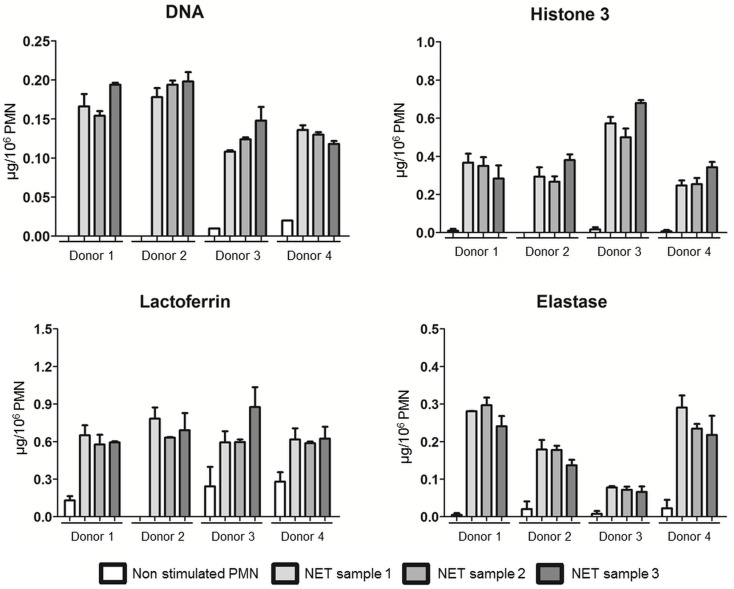
**NET preparation and characterization are reproducible**. dsDNA, H3, LF, and ELA concentrations of A23187-NET samples from four healthy donors. Each fresh blood sample was divided in three parts, and three independent NET isolations, and characterizations were done. Data are means ± SEM (*n* = 3). Results are expressed in μg/10^6^ PMN.

As interindividual variations were observed, we assessed reproducibility testing in additional four different healthy donors. We confirmed that the NET composition of the four donors was quite different in terms of dsDNA, H3, LF, and ELA levels. Interestingly, three independent NET samples were prepared for each donor that gave similar results as evidenced in Figure 6 (dsDNA, H3, LF, and ELA levels).

### Isolated NETs retain their microbicidal activity

**Figure 7:**
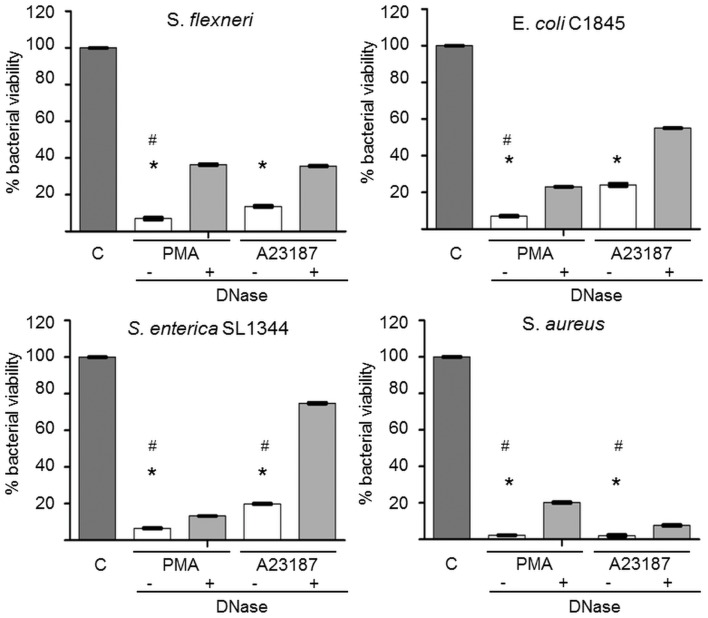
**Alu-I-derived NETs retain microbicidal activity partially dependent on DNA integrity**. *S. aureus, E. coli* C1845, *S. flexneri*, and *S. enterica serovar* Typhimurium SL1344 were incubated for 45 min in the presence of isolated NETs obtained after *Alu*I treatment of A23187- or PMA-activated PMN. In some experiments, NET samples were pretreated with DNase to dismantle NETs. Bacterial viability was measured by a colony count assay (CFU/mL). Results are expressed as percentage bacterial viability, calculated from CFU/mL values of bacteria exposed to NETs relative to bacteria not exposed to NETs (control tube = C). *p < 0.05 versus bacterial viability in the absence of NETs. *p < 0.05 versus DNase-treated NETs.

Finally, we examined whether isolated NETs (obtained after PMA or A23187 stimulation) retained their bactericidal activity. We used four bacterial species previously shown by us and others to be killed by NETs (Brinkmann et al., 2004; Li et al., 2010; Marin-Esteban et al., 2012). *S. flexeneri* strain M90T, *S. enterica* serovar typhimurium strain SL1344, *S. aureus*, and the wild-type *E. coli* strain C1845 were exposed to isolated NETs for 45 min. As shown in Figure 7, more than 90 and 70% of the bacteria, respectively, were killed by PMA-derived and A23187-derived NETs. In both cases this effect was partially dependent on DNA integrity, as DNase pretreatment of NETs increased bacterial viability; however, this effect of DNase was always significant for PMA-derived NETs but only against *S. aureus* and *S. enterica* SL1344 for A23187-derived NETs (*p* < 0.05).

## Discussion

Neutrophil extracellular traps have been extensively studied since their discovery in 2004, but many aspects of their composition, roles, regulation, and involvement in disease states remain unclear and even controversial. In particular, the beneficial or detrimental effect of NETs might be determined by the intensity of the netosis response, and better knowledge of netosis could thus help to design NET-targeting therapies (Brinkmann and Zychlinsky, 2012; Cheng and Palaniyar, 2013; Simon et al., 2013). However, standardized procedures are needed to isolate NETs suitable for such studies. Brinkmann et al. (2012) recently described a simple automated method of NET quantification. Here, we describe a novel approach to recover, characterize and quantify large functional NETs produced by human neutrophils.

The first step was to identify an appropriate NET inducer. Calcium ionophore A23187 was chosen because it was as potent as PMA, did not induce cell death and can be efficiently removed by washing. Moreover, A23187 has been shown to trigger netosis of neutrophils from zebrafish (Palic et al., 2007) and of the myeloid leukemia cell line HL-60 (Wang et al., 2009). The second step was to determine the most appropriate nuclease for NET digestion. As DNase might inhibit the microbicidal effect of NETs (Urban et al., 2009) and as MNase is known to generate only small NET structures (Liu et al., 2012; Saffarzadeh et al., 2012), we chose Alu-I, which yielded heterogeneous populations of dsDNA fragments ranging from 0.5 kbp to more than 15 kbp. NETs of various sizes, including large fragments, are likely to be more physiologically relevant than small NETs obtained with DNAse or MNase.

In addition to DNA, we quantified the proteins LF, ELA, and H3 in isolated NETs. LF and ELA are important mediators of netosis, as they are involved in pathogen destruction and cell activation or toxicity. Our NET samples also contained citrullinated H3 (H3cit), one the main signatures of netosis (Li et al., 2010; Neeli and Radic, 2012). During netosis, histones are degraded by ELA and undergo citrullination of lysine residues by the enzyme PAD4 (Urban et al., 2009; Papayannopoulos et al., 2010; Neeli and Radic, 2012), both processes contributing to the lower molecular weight of H3 that we observed on NET immunoblots. As our aim was to develop a standardized procedure for isolating human NETs, we compared the composition of NETs obtained with neutrophils from 10 healthy volunteers. Interestingly, NETs from the different donors contained differing amounts of LF, ELA, H3, H3cit, and DNA. This interindividual variability may be related to variations in the rate of netosis, as reported by Urban et al. (2009) and more recently confirmed by Brinkmann et al. (2012). Interindividual variations in both the netosis rate and NET composition should therefore be taken into account when assessing netosis in clinical situations. Reproducibility of NET preparation and characterization was validated, suggesting that the interindividual variability in NET composition was related to donor variation, and not to technical bias.

We found that our isolated NETs retained their microbicidal activity, which reached about 70% with four different bacterial strains. This bactericidal effect was only partially reversed by DNase, suggesting that NET structure is important but that soluble microbicidal mediators are also involved. NET-mediated killing appears to be related to electrostatic interactions, histones, and high local concentrations of antimicrobial peptides such as proteases, defensins, and cathelicidin (Brinkmann and Zychlinsky, 2012; Simon et al., 2013). In our model, these peptides appeared to retain their antimicrobial capacity. However, NET-mediated antimicrobial activity is controversial (Nauseef, 2012; Simon et al., 2013). Indeed, recent studies suggest that NETs might simply have a bacteriostatic effect, through physical containment (Menegazzi et al., 2012). However, the bacterial killing observed with our NET preparations, which are smaller than PMN-associated non-digested NETs *in vivo*, suggests that physical containment might not be essential for NET antibacterial activity. This question could be addressed using our NET model.

Neutrophil extracellular traps can also damage host tissues by releasing proteases such as ELA (Marin-Esteban et al., 2012; Saffarzadeh et al., 2012; Villanueva et al., 2012). As our NET preparations exhibited ELA activity, our model would be suitable for studying NET-induced host cell cytotoxicity (Metzler et al., 2010; Papayannopoulos et al., 2010). Saffarzadeh et al. (2012) recently showed that MNase-derived isolated NETs were cytotoxic for endothelial cells via histone- and MPO-dependent mechanisms. Conversely, the cytotoxic activity of non-isolated NETs cocultured with endothelial cells can be partially down regulated by MNase treatment (Villanueva et al., 2012). These results illustrate the potential interference arising from PMN-derived elements when NETs are not isolated, and underline the importance of standardizing NET isolation.

Neutrophil extracellular traps have recently been shown to modulate various immune cell functions, activating plasmacytoid dendritic cells (Villanueva et al., 2012) and myeloid dendritic cells (Sangaletti et al., 2012), inducing autoimmune responses (Sangaletti et al., 2012; Simon et al., 2013), and priming T lymphocytes by lowering the TCR activation threshold (Tillack et al., 2012). These findings were made using small MNase-digested NETs. The importance of the integrity of larger NET structures in other NET effects on adaptive immune responses could be examined using our standardized model.

In summary, we describe a new method for isolating large NETs bearing the main characteristic components of NETs and retaining their antibacterial and protease activities. Isolated NETs are potentially an important tool for describing new effects of NETs and for dissecting, both *in vitro* and in animal models, their contribution to physiological and pathological processes, with little or no interference from other PMN components.

## Conflict of Interest Statement

The authors declare that the research was conducted in the absence of any commercial or financial relationships that could be construed as a potential conflict of interest.
